# Genetic variant for behavioral regulation factor of executive function and its possible brain mechanism in attention deficit hyperactivity disorder

**DOI:** 10.1038/s41598-018-26042-y

**Published:** 2018-05-16

**Authors:** Xiao Sun, Zhaomin Wu, Qingjiu Cao, Ying Qian, Yong Liu, Binrang Yang, Suhua Chang, Li Yang, Yufeng Wang

**Affiliations:** 1 Peking University Sixth Hospital (Institute of Mental Health), National Clinical Research Center for Mental Disorders & Key Laboratory of Mental Health, Ministry of Health (Peking University), 51 Huayuan Bei Road, Beijing, 100191 China; 2 CAS Key Laboratory of Mental Health, Institute of Psychology, 16 Lincui Road, Beijing, 100101 China; 3 College of Life Sciences and Bioengineering, Beijing Jiaotong University, 3 Shang Yuan Cun, Beijing, 100044 China; 4 Shenzhen Children’s Hospital, 7019 Yitian Road, Shenzhen, 518038 China; 5 Department of Psychology, University of Chinese Academy of Sciences, 19 Yuquan Road, Beijing, 100049 China

## Abstract

As a childhood-onset psychiatric disorder, attention deficit hyperactivity disorder (ADHD) is complicated by phenotypic and genetic heterogeneity. Lifelong executive function deficits in ADHD are described in many literatures and have been proposed as endophenotypes of ADHD. However, its genetic basis is still elusive. In this study, we performed a genome-wide association study of executive function, rated with Behavioral Rating Inventory of Executive Function (BRIEF), in ADHD children. We identified one significant variant (rs852004, *P* = 2.51e-08) for the overall score of BRIEF. The association analyses for each component of executive function found this locus was more associated with inhibit and monitor components. Further principle component analysis and confirmatory factor analysis provided an ADHD-specific executive function pattern including inhibit and monitor factors. SNP rs852004 was mainly associated with the Behavioral Regulation factor. Meanwhile, we found the significant locus was associated with ADHD symptom. The Behavioral Regulation factor mediated its effect on ADHD symptom. Functional magnetic resonance imaging (fMRI) analyses further showed evidence that this variant affected the activity of inhibition control related brain regions. It provided new insights for the genetic basis of executive function in ADHD.

## Introduction

Attention deficit hyperactivity disorder (ADHD) is a common psychiatric disorder characterized by age-inappropriate deficiency in sustained attention and/or hyperactive, impulsive behaviors^1^. The genetic epidemiological studies revealed gene variants constituted the primary etiology of ADHD, with a heritability estimated to be 0.76^2^. Candidate genes were involved in the biosynthesis, release, transmission and metabolism of neurotransmitters^3^, while genome-wide association studies^4–7^ suggested genes contributing to the brain development. The complex genetic architecture of ADHD is still far from fully illustrated^2,8^.

One of the reasons for the complexity of ADHD is that it is a heterogeneous disorder. The same clinical presentation of inattention, hyperactivity and impulsivity may have different etiological contribution. Identification of the genetic contributions to ADHD is likely complicated by phenotypic and genetic heterogeneity, low penetrance, and limited statistical power. One way to enhance power for genetic discovery is to reduce heterogeneity by use of endophenotypes. Presently, the most common endophenotypes under consideration are neuropsychological markers of executive function^9,10^. Executive function (EF) is a high order cognitive function that provides people the capacity to change and adjust behaviors according to the shifting demands of the complex environment^11^. Executive dysfunction has been found in many psychiatric disorders, such as bipolar disorder^12^, depression^13^, schizophrenia^14^, autism^15^ and ADHD^16^. Candidate genes involved in the neurotransmitter system, including dopaminergic, noradrenergic, serotonergic and cholinergic, have been reported to be associated with some component of executive functions^17^. Yang *et al*. identified one significant locus for inhibition in ADHD by using genome-wide association study^18^.

In this study, we performed a genome-wide association analysis on the global score of executive function using the Behavior Rating Inventory of Executive Function (BRIEF) and further analyzed the association of the significant locus with each component of executive function and ADHD symptoms. Furthermore, we analyzed the function of associated locus via neuroimaging study in human and its underlying molecular mechanism contributing to brain function and behavior. The findings may provide new insights into the pathological mechanisms of ADHD.

## Results

### Correlation between executive function and ADHD symptoms

We collected three dimensional symptoms for the patients, namely inattention (CDISatt), hyperactivity-impulsivity (CDIShi) and overall assessment (CDISall). We calculated the correlations between the BRIEF scales and three symptom traits (as shown in Table 1). All BRIEF scales were significantly correlated with inattention symptom, but only eight of them were significantly correlated with hyperactivity-impulsivity symptom (Total score, Behavior Regulation Index, Metacognition Index, Inhibit, Emotional Control, Working Memory, Organization of Materials, Monitor), and nine with overall assessment (Total score, Behavior Regulation Index, Metacognition Index, Inhibit, Emotional Control, Working Memory, Plan/Organize, Organization of Materials, Monitor).

**Table 1:** Correlations between the BRIEF scales and the three symptom traits from the ADHD Rating scale-IV-patient report.

	CDISatt		CDIShi		CDISall	
	Cor.	Sig.	Cor.	Sig.	Cor.	Sig.
Total	0.3748	<**2.2e-16**	0.1825	**2.05E-05**	0.2406	**1.60E-08**
BRI	0.2206	**2.37E-07**	0.2033	**2.00E-06**	0.2283	**8.63E-08**
MI	0.4275	<**2.2e-16**	0.1317	**2.21E-03**	0.2053	**1.58E-06**
IB	0.2633	**5.55E-10**	0.2916	**5.25E-12**	0.3177	**4.43E-14**
SFT	0.1175	**6.38E-03**	0.0292	5.00E-01	0.0496	2.51E-01
ECTRL	0.1348	**1.72E-03**	0.1225	**4.45E-03**	0.1373	**1.41E-03**
INIT	0.2619	**6.87E-10**	0.0282	5.14E-01	0.0764	7.67E-02
WM	0.4393	<**2.2e-16**	0.1186	**5.87E-03**	0.1956	**4.87E-06**
PO	0.3291	**4.67E-15**	0.0730	9.05E-02	0.1322	**2.12E-03**
OM	0.3644	<**2.2e-16**	0.1264	**3.32E-03**	0.1893	**9.85E-06**
MONI	0.2790	**4.45E-11**	0.1891	**1.01E-05**	0.2283	**8.58E-08**

R function cor.test is used for this analysis. Cor. is correlation; Sig. is significance. BRI, Behavior Regulation Index; MI, Metacognition Index; IB, Inhibit; SFT, Shift; ECTRL, Emotional Control; INIT, Initiate; WM, Working Memory; PO, Plan/Organize; OM, Organization of Materials; MONI, Monitor.

### SNPs associated with executive function components in BRIEF

**Figure 1:**
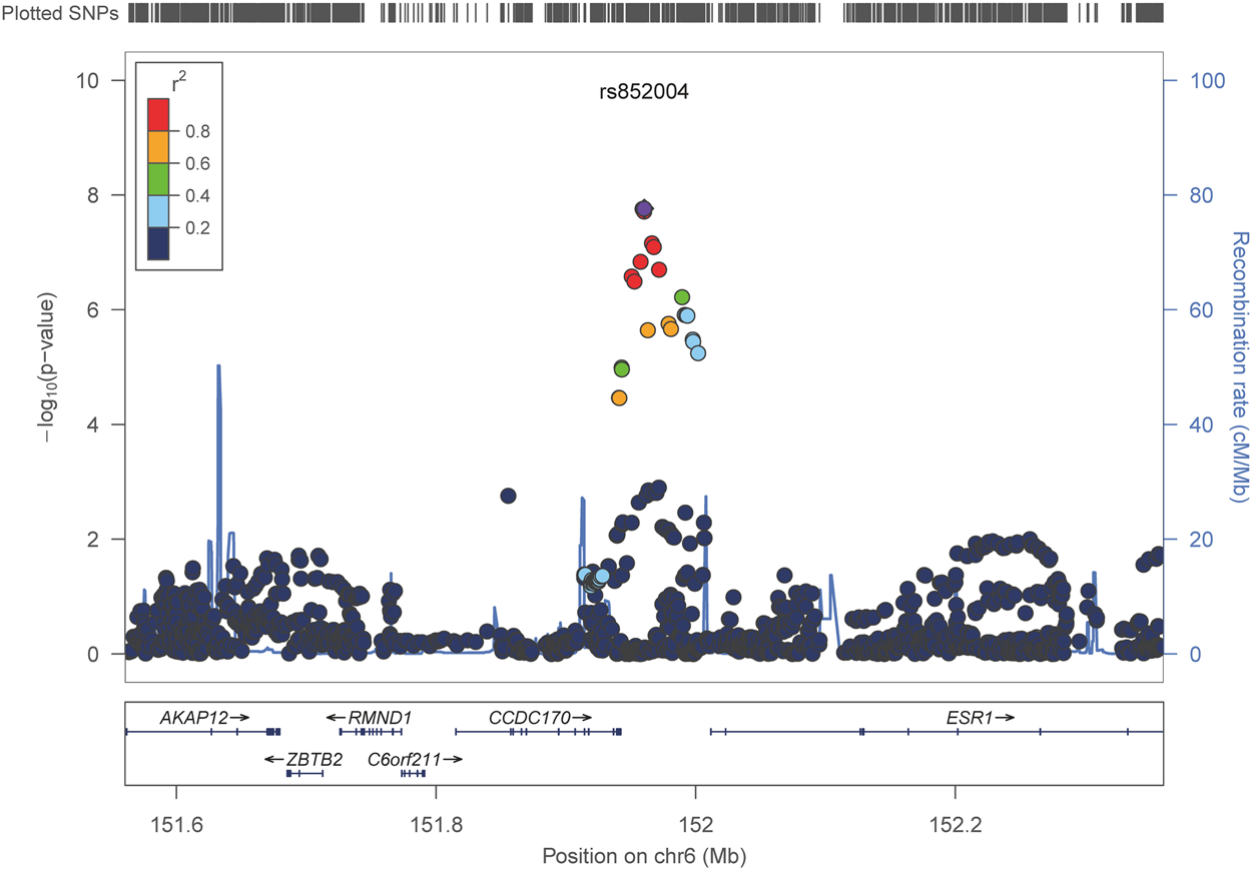
Regional plot for the significant locus with total score in BRIEF. LocusZoom (http://locuszoom.org/) was used to generate the plot using the association analysis result after imputation.

Firstly, we analyzed the association of SNPs with the global score of BRIEF. We identified one significant SNP (rs852004, *P* = 2.51e-08, A1 = A, frequency = 0.08, BETA (95% CI) = −11.84 (−15.94, −7.734)) located in 6q25.1. The A allele carriers had smaller BRIEF score, which means better executive function. Regional plot for this locus was shown in Fig. 1. This locus was within the upstream of ESR1, RMND1, ZBTB2 and downstream of CCDC170 and C6orf211. Next, we analyzed the association of this significant SNP with each subscale of BRIEF. As shown in Table 2, this SNP is the first top associated SNP with the BRI (Behavioral Regulation Index for Inhibit, Shift and Emotional Control) and MI (Metacognition Index for Initiate, Working Memory, Plan/Organize, Organization of Materials, Monitor). The association with BRI was mainly from Inhibit (top one significant SNP), while the association with MI was mainly from Monitor (top one significant SNP).

**Table 2:** Phenotype information and the association result of the significant SNP rs852004 in each scale of BRIEF.

Name	Phenotype	Mean (SD)	*P*-value	Corrected *P*-value^a^	Effect Size (95% CI)	Rank
Total	Total score for all 8 scales of BRIEF	151.98 (19.56)	2.51E-08	2.51E-08	−11.84 (−15.94, −7.734)	1
IB	Inhibit	20.04 (4.59)	9.52E-08	1.14E-06	−2.642 (−3.599, −1.685)	1*
SFT	Shift	12.89 (2.75)	3.58E-03	4.30E-02	−0.8729 (−1.458, −0.2881)	2326
ECTRL	Emotional Control	17.27 (4.48)	6.92E-04	8.30E-03	−1.661 (−2.615, −0.7071)	418
INIT	Initiate	15.69 (2.96)	5.52E-04	6.62E-03	−1.118 (−1.749, −0.4876)	333
WM	Working Memory	23.06 (3.17)	3.38E-04	4.06E-03	−1.244 (−1.92, −0.568)	189
PO	Plan/Organize	27.99 (3.98)	3.39E-06	4.07E-05	−2.009 (−2.848, −1.17)	2
OM	Organization of Materials	14.58 (2.61)	5.38E-03	6.46E-02	−0.7922 (−1.348, −0.2366)	3327
MONI	Task-Monitor	20.46 (2.75)	5.05E-07	6.06E-06	−1.496 (−2.072, −0.9195)	1*
BRI	Behavioral Regulation Index for IB, SFT and ECTRL	50.2 (9.68)	9.11E-07	1.09E-05	−5.176 (−7.218, −3.134)	1
MI	Metacognition Index for INIT, WM, PO, OM and MONI	101.78 (12.16)	4.75E-07	5.70E-06	−6.66 (−9.22, −4.099)	1
PC1	Principle component containing IB, INIT, WM, PO, OM and MONI	N.A.	2.013E-07	2.42E-06	−0.5655 (−0.7759, −0.355)	1
PC2	Principle component containing SFT and ECTRL	N.A.	6.194e-05	7.43E-04	−0.4364 (−0.6483, −0.2246)	32

SD: standard deviation. The association analysis for PC1 and PC2 were performed for the normalized value, so the mean and SD were not available. Rank is the rank of SNP rs852004 in the genome-wide association result for each BRIEF scale. The rank marked with * means the rank is for rs6908732, which is in high LD with rs852004 (*r*^*2*^ = 0.75). A1 = A, allele frequency = 0.08.

^a^Since we firstly performed the GWAS for the total score of BRIEF, the P-value for Total didn’t need multiple correction. Then, we checked the association of the significant SNP in 8 scales, two indexes and two PCs. The corrected P-value for them were obtained by multiplying 12.

### Association of the significant SNP with principle component of BRIEF

The component plot in the rotated space and rotated component matrix from the PCA for the eight subscales of BRIEF was shown in Supplementary Fig. S1. Two components were detected (eigen value >1 in scree plot). The first component is related with Inhibit (IB), Initiate (INIT), Working Memory (WM), Plan/Organize (PO), Organization of Materials (OM) and Monitor (MONI); the second component is related with Shift (SFT) and Emotional Control (ECTRL). Furthermore, we extracted the two components values and conducted association analysis for the two components. As shown in Table 2, rs852004 was the top one SNP in the association result of component 1, while, the SNP ranked 32 in the association result of component 2.

**Figure 2:**
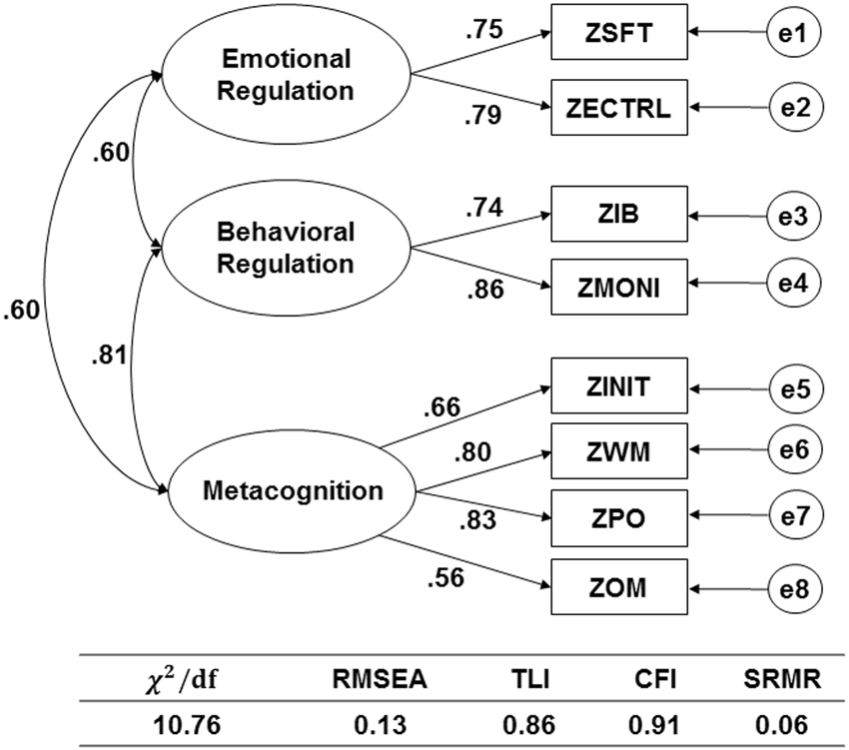
The 3-factor model for the confirmatory factor analysis of the BRIEF data. The fit parameters for the model was shown below the model.

Since the components from PCA is slightly different with the definition of the two indexes in BRIEF (BRI and MI), we further performed confirmatory factor analysis (CFA) for the two candidate models. The Model A is based on the definition of BRIEF indexes: one group constituted of IB, SFT and ECTRL, the other group constituted of INIT, WM, PO, OM and MONI (see Supplementary Fig. S2a). The Model B is based on the PCA result: one group constituted of IB, INIT, WM, PO, OM and MONI, the other group constituted of SFT and ECTRL (see Supplementary Fig. S2b). The result showed the Model B is more suitable for our samples. Next, we further checked a 3-factor model as shown in Fig. 2 (Model C), in which, we grouped IB and MONI into one subgroup. The CFA result showed the 3-factor model was more suitable for our data than the two 2-factor models.

### Association of the significant SNP with ADHD symptom

SNP rs852004 was not significant in our Chinese ADHD case-control GWAS (*P* = 0.1619)^4^. Association analysis for the significant SNP rs852004 with ADHD symptoms was conducted to further validate the contribution of the significant SNP rs852004. Among the 550 samples, 533 samples have both symptoms data and genotype data for SNP rs852004. The analysis showed it was significantly associated with the total assessment (*P* = 0.0163, A1 = A, BETA = −0.5733), which denoted the ADHD symptom of A carriers was better.

Furthermore, we examined the role of each BRIEF scale as a mediator to mediate the association between rs852004 with the ADHD symptom. We used the model 4 in PROCESS^19^ to bootstrap the sampling distribution of the indirect effect (where the indirect effect is the reduction in the strength of the SNP/symptom association that is due to the executive function). The indirect effect of rs852004 on ADHD total symptom score (CDISall) through each BRIEF scale was shown in Supplementary Table S1. The result showed all BRIEF scales have significant indirect effect on ADHD symptom. The mediation effect was different to zero even at the lower bound of the confidence interval. Furthermore, the effect of Inhibit (IB), Monitor (MONI), Working Memory (WM) and Plan/Organize (PO) were bigger among the eight scales. These data showed that SNP rs852004 accounts for significant variation in ADHD symptom, in part through the effects of the SNP on the intermediate phenotype of executive function.

### Function validation of associated SNP rs852004 by resting-state fMRI

**Figure 3:**
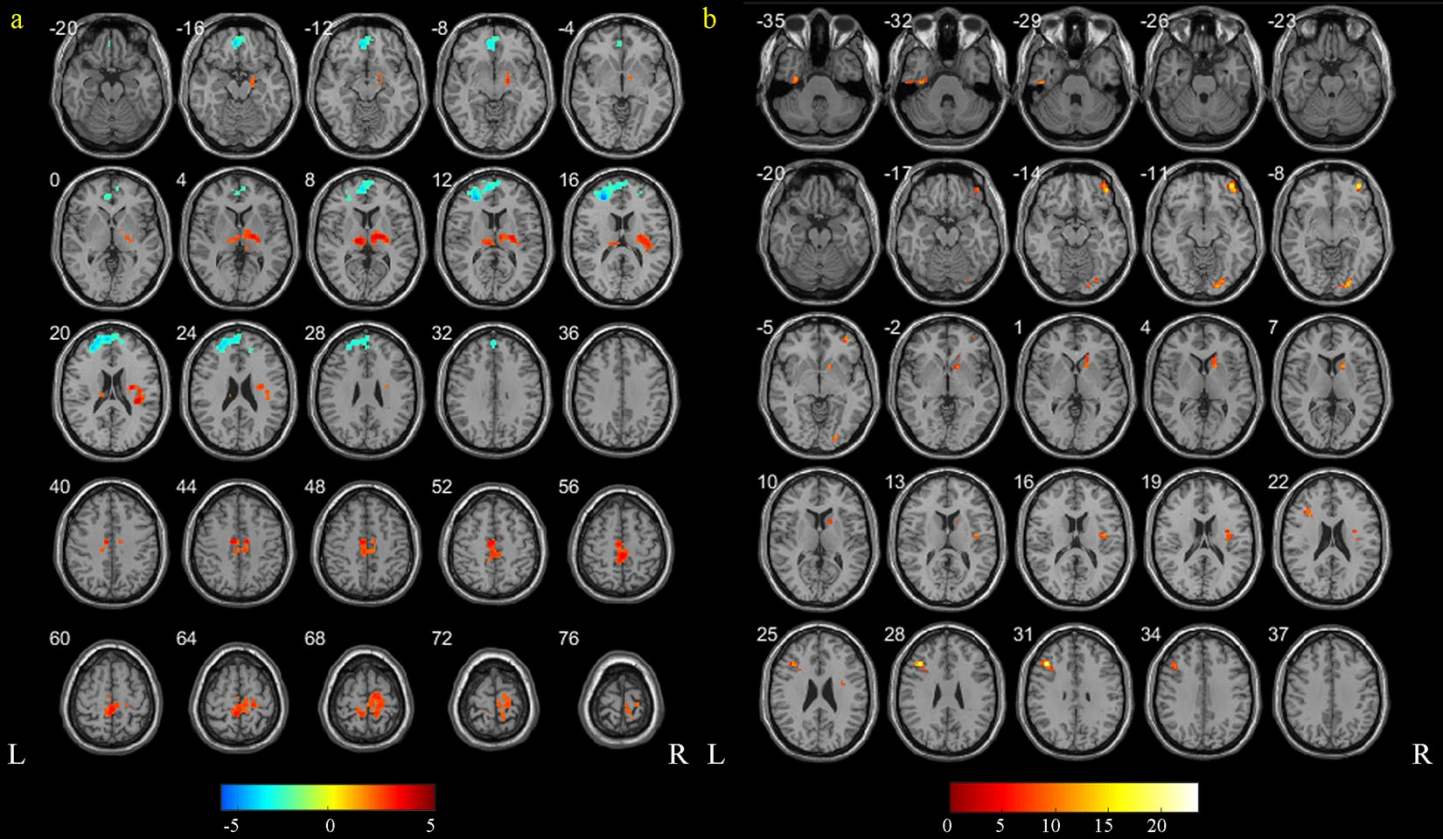
Regions exhibiting differences in ReHo. (**a**) regions showed the differences between ADHD individuals and controls (T values, *P* < 0.01). (**b**) regions showed the interaction of rs852004 and ADHD (F values, *P* < 0.01).

Among 50 ADHD patients, 42 are G homozygotes for rs852004; among 66 control children, 57 are G homozygotes for rs852004. Compared with healthy controls, reduced Regional Homogeneity (ReHo) was uncovered in ADHD in dorsolateral prefrontal cortex, medial prefrontal cortex and increased ReHo in thalamus, anterior cingulate cortex, and anterior insular cortex (Fig. 3a, T values, *P* < 0.01, cluster size above 202 voxels). The interaction of diagnosis and genotype was found in six clusters, including right orbitofrontal cortex, dorsal striatum, insula, lingual cortex, left inferior frontal cortex, inferior temporal cortex (Fig. 3b, F values, *P* < 0.01, cluster size above 31 voxels). In term of the simple effect of rs852004, compared to G homozygote, no increased or reduced ReHo was discovered after multiple comparison in either ADHD or control group.

### Regulatory feature and expression quantitative trait loci (eQTL) analysis of associated SNP rs852004

The significant locus we identified was within the upstream of ESR1, RMND1, ZBTB2 and downstream of CCDC170 and C6orf211 (as shown in Fig. 1). It may affect the nearby genes by regulatory mechanism. We obtained nine SNPs with high LD with rs852004 and checked the related regulatory features of the SNPs by searching rVarBase and RoadMap data. In the LD block of the significant locus, there is only some weak enhancer, DNase hyperactivity site and heterochromatin signal in RoadMap (upper panel of Supplementary Fig. S3, only neuronal cells and brain tissues were shown). rVarBase showed these SNPs were located in chromatin interactive region, which targeted genes *RMND1*, *C6orf*2*11*, *CCDC170* and *ESR1*. eQTL data searching found rs852004 and rs6908732 could regulate the expression of *ZBTB*2 in the cerebellum of bipolar disorder^20^, and rs852004 could regulate the expression of *RMND1* in human prefrontal cortex^21^. We further searched the expression profiles of these genes in human brain tissues in BRAINEAC database and performed eQTL analyses to elucidate whether SNP rs852004 influences the genes expression in the brain. *CCDC170* was not found in this database, and other four genes (*RMND1*, *C6orf211*, *ESR1*, *ZBTB2*) are expressed in various brain regions, with the highest transcript level in cerebellar cortex (*RMND1*), thalamus (*C6orf211* and *ZBTB2*), and inferior olivary nucleus (*ESR1*). We found significant association between rs852004 with these genes separately in thalamus (*RMND1*, *P* = *0.0081*), cerebellar cortex (*C6orf211*, *P* = *0.024*; *ESR1*, *P* = *0.0021*), frontal cortex (*ESR1*, *P* = *0.049*). All the eQTL data were summarized in Table 3.

**Table 3:** The eQTL data summary of significant SNP rs852004.

Source	Tissue	SNP	Gene	*P-value*
SCAN	cerebellum	rs852004	ZBTB2	N.A.
SCAN	cerebellum	rs6908732	ZBTB2	N.A.
Liu C *et al*.^21^	prefrontal cortex	rs852004	RMND1	4.42E-03
BRAINEAC	FCTX	rs852004	ESR1	4.90E-02
BRAINEAC	THAL	rs852004	RMND1	8.10E-03
BRAINEAC	HIPP	rs852004	RMND1	4.70E-02
BRAINEAC	CRBL	rs852004	C6orf211	2.40E-02
BRAINEAC	CRBL	rs852004	ESR1	2.10E-03

FCTX, frontal cortex; THAL, thalamus; HIPP, hippocampus; CRBL, cerebellar cortex. N.A. is not available.

## Discussion

Dysregulation of executive function is a key deficit of ADHD. In this study, a genome-wide association study was conducted to explore genetic loci associated with impaired executive function evaluated by BRIEF in ADHD. A significant SNP rs852004 was found to be associated with BRIEF behavioral evaluation of executive function. We further checked the association of the significant SNP with each subscale of BRIEF, and found rs852004 was more associated with Inhibit and Monitor. According to the primary description of BRIEF^22^, Inhibit and Monitor belong to different indexes. Our principle component analysis found different pattern for the eight scales of BRIEF in ADHD patients. In this population, inhibit was more correlated with the metacognitive index, which was further validated by our confirmatory factor analysis result.

By using CFA, we built a three-factor model for BRIEF (Fig. 2). Normally, the model with TLI and CFI > 0.8, χ^2^/df < 5, RMSEA and SRMR < 0.1 were considered to be acceptable. In Model C, TLI, CFI and SRMR satisfied the criteria, but χ^2^/df and RMSEA is larger than the threshold. χ^2^/df is proportioned with the sample size. We tried to use half of the sample size to fit the model, and the χ^2^/df became almost half of the current value. In addition, both χ^2^/df and RMSEA were related with the correlation between different groups. Since Inhibit had significant correlation with Working Memory, Initiate and Emotional Control scales, it is one of the reasons for the larger RMSEA^23^. Gioia *et al*. had described a 3-factor model for BRIEF by parsing Monitor into Self-Monitor and Task-Monitor^23^. The 3-factor model included Behavioral Regulation factor (Inhibit, Self-Monitor), Emotional Regulation factor (Emotional Control, Shift), and Metacognition factor (Initiate, Working Memory, Plan/Organize, Organization of Materials, Task-Monitor). The Model C we established based on the association result of rs852004 was similar with their 3-factor model although the Monitor was not parsed in our model. Based on this model, SNP rs852004 was mainly associated with Behavioral Regulation factor. Remarkably, in our model, Behavioral Regulation factor is more correlated with Metacognition factor, but not Emotional Regulation factor as Gioia *et al*.’s 3-factor model^23^. One possible explanation for this is disorder-specific executive function profile. This model is more consistent with Barkley’s view of executive function in ADHD^24^: inhibitory control having a unique and separable role in executive function; inhibition is more primary and plays an underlying role that enables other functions including working memory, emotional regulation and goal directed analysis and synthesis in problem-solving. Furthermore, in ADHD, the underlying “enable” role of inhibition may have more apparent effect on metacognition factor, including working memory, monitor and plan/organize. The indirect mediation effect of rs852004 on ADHD symptom through each BRIEF scale (see Supplementary Table S1) also supported this, which showed inhibit, monitor, working memory and plan/organize had bigger effect on ADHD overall symptom.

Furthermore, our fMRI data on ADHD and controls suggested specific inhibitory activation in dorsal lateral and medial prefrontal cortex, right insular, putman and supplementary motor area which was overlapped with previous literature reports^25^. The fMRI data combined with genetic data showed that the associated genetic locus might affect the brain function. The interaction effect of gene and diagnosis was uncovered in brain regions of dorsolateral prefrontal striatum circuit, which was known as the basis of inhibition and in accordance with previous meta-analysis^26^. It suggested that the associated SNP might have modulating effect on the function of brain region responsible for executive inhibition.

SNP rs852004 and its LD-proxy rs6908732 were suggested to be associated with schizophrenia^27^, major depressive disorder^28^ and bipolar disorder^29^, all of these disorders had executive function deficits^30–32^. Regulatory feature analyses found this locus was within the chromatin interactive region, which targeted genes *RMND1*, *C6orf211*, *CCDC170* and *ESR1*. SNP in *ESR1* also predicted intracranial volume^33^. Meanwhile, eQTL data showed SNP rs852004 regulated the expression of *ZBTB2* in cerebellum of bipolar disorder patients and human prefrontal cortex. *ZBTB2* is a regulator of p53 pathway^34^. The homolog gene of *ZBTB2* – *ZBTB20* has been reported to modulate the sequential generation of neuronal layers in developing cortex^35^. It is also reported that hyper methylation in the *ZBTB20* gene is associated with major depressive disorder^36^. But the report about the association of *ZBTB2* with psychiatric disorder was not much. In consist with our fMRI analysis, our eQTL analyses showed that the SNP rs852004 influenced *ZBTB2*, *RMND1*, *C6orf211* and *ESR1* expression in many brain regions especially frontal cortex (*ESR1*) and thalamus (*RMND1*) which constitutes dorsolateral prefrontal striatum circuit, which is the basis of inhibition, including response inhibition and interference inhibition^37^. We hypothesized that SNP rs852004 influenced these genes expression in consequence to affect the executive function, especially inhibition, and ADHD symptoms.

The detailed molecular mechanism of the significant locus on inhibition related brain function and behavior need further validation study. In consideration of the complexity of cognition and behavior, it’s likely that more genes are still needed to be discovered in larger samples, and more aspects of executive function should be discussed.

## Methods

### Genome wide association study samples

Totally, 550 subjects finished the Behavior Rating Inventory of Executive Function (BRIEF) (470 boys, 80 girls) aged between 6 and 16 years (average 9.77 ± 2.44 years). All participants in this study were recruited from the Child and Adolescent Psychiatric Outpatient Department of Peking University Sixth Hospital. All cases met DSM-IV ADHD diagnostic criteria. A clinical diagnosis was first made by a senior child and adolescent psychiatrist based on the ADHD Rating Scale-IV (ADHD-RS-IV) completed by parents (and teacher when available), and then confirmed by semi-structured interview using the Chinese version of the Clinical Diagnostic Interview Scale^38^. Those comorbidities with major neurological or psychiatric disorders including epilepsy, schizophrenia, pervasive development disorder, and mental retardation (IQ < 70) were excluded.

### Resting-state fMRI study samples

A total of 50 ADHD patients (47 boys, 3 girls, 42 G homozygotes for rs852004) and 66 control children (37 boys, 29 girls, 57 G homozygotes for rs852004) aged between 8 and 16 years were enrolled from the Child and Adolescent Psychiatric Outpatient Department of Peking University Sixth Hospital in this section. All the participants were right-handed. The ADHD samples are part of the above ADHD samples for BRIEF. The controls were also interviewed to ensure that they were free of any Axis I psychiatric disorders. This study was approved by Institutional Review Board of Peking University Sixth Hospital and written informed consent was signed by parents.

### Executive function test and three dimensional symptoms in ADHD patients

We assessed the executive function using BRIEF, an 86-item questionnaire designed for parents of children aged 5–18 years to assess executive function behaviors. In the questionnaire, the parent responds whether their child exhibits problems with specific behaviors: Never, Sometimes or Often, scored as 1, 2, or 3, respectively. The questionnaire includes two domains: Metacognition Index (MI) comprising of five subscales, *i.e*. Initiate (INIT), Working Memory (WM), Plan/Organize (PO), Organization of Materials (OM) and Monitor (MONI); while Behavior Regulation Index (BRI) comprising of three subscales, *i.e*. Inhibit (IB), Emotional Control (ECTRL) and Shift (SFT)^23,39^. The score of each subscale equals to the sum of the scores of all items belonged to that subscale; the scores of two indexes equals to the sum of the scores of all subscales belonged to the index. The score of the Global Executive Composite equals to the sum of the scores of the two indexes. All these traits and their name abbreviation used in this study were shown in Table 2. Besides the BRIEF, we have collected three dimensional symptoms, namely inattention (CDISatt), hyperactivity-impulsivity (CDIShi) and overall score (CDISall), for the patients according to the Clinical Diagnostic Interview Scale^40^. CDISatt denotes the inattention symptom score, CDIShi denotes the hyperactivity-impulsivity symptom score, and CDISall denotes the sum of CDISatt score and CDIShi score.

### Genotyping and quality control

Genomic DNA was extracted from peripheral blood using Omega DNA extraction Kit (Omega Bio-tek Inc., Doraville, GA). Genotypes were obtained using the Affymetrix6.0 array at CapitalBio Ltd. (Beijing) using the standard Affymetrix protocol. The Affymetrix6.0 array included 906,600 SNP probes. After mapping them to SNPs with #rs, 653,428 SNPs were left. For quality control, the individuals (1) with per-individual autosomal heterozygosity >5 s.d. away from the mean, (2) having no age or IQ information, (3) per-individual call rate <95% and (4) with relatives having genome identity PI_HAT ≥ 0.185 were removed. Then, the remaining samples were assessed for population stratification using Principal Component Analysis (PCA) implemented in EIGENSOFT4.2^41,42^. Tracy-Widom test was employed to detect significant eigenvectors (*P* < 0.05). Only the first eigenvector (eigenvector 1) was significant, which was used as a covariate in the subsequent statistical analysis. When controlling for the quality of SNPs, we removed SNPs if (1) per-SNP call rate <98%, (2) Hardy-Weinberg equilibrium test *P* < 0.001, (3) MAF < 1%. Totally, 644,166 autosomal SNPs were analyzed in 547 ADHD patients after quality control.

### Resting-state fMRI data collection

Resting-state fMRI data acquisition, preprocessing and quality control detailed is described in the supplementary material.

### Statistical Analyses

#### Genome wide association test and imputation

Association analysis for each quantitative trait was conducted using an additive model in linear regression in PLINK^43^ with age, IQ, sex and eigenvector 1 of PCA as covariates. Two-sided *P* < 5 × 10^−8^ was considered as genome-wide significance. We used MACH-admix 1.0^44^ to impute non-genotyped SNPs using the ASN data (286 individuals) from the 1000 Genomes Project Integrated Phase 1 Release^45^ as the reference panel. Imputed SNPs with squared correlation between imputed and true genotypes (rsq) <0.6 or SNPs with MAF < 0.01 were removed. Association analysis after imputation was done using mach2qtl^46^.

#### Principle component analysis and confirmatory factor analysis

All scores of the eight scales of BRIEF were normalized prior the principle component analysis (PCA) and confirmatory factor analysis (CFA). The PCA was conducted in SPSS. CFA for the several candidate models of BRIEF was conducted using AMOS. The maximum likelihood method was used. Fit of all models was evaluated using several indexes, including the χ^2^/df value, the root mean square error of approximation (RMSEA), the Comparative Fit Index (CFI), Tucker-Lewis Index (TLI) and standardized root mean-square residual (SRMR). Smaller χ^2^/df, RMSEA and SRMR values and larger CFI and TLI values indicate a better fit.

#### Resting-state fMRI data analysis

Individual ReHo map was generated in DPABI by calculating the Kendall coefficient of concordance (KCC) of the time series of a given voxel with those of its neighbors (26 voxels) in a voxel-wise way^47^ and the inclusive threshold for each voxel was set to *P* < 0.01. The following statistic analysis was conducted in SPM (http://www.fil.ion.ucl.ac.uk/spm/software/spm12/). A T test was applied to identify the differences between ADHD and control, and then a full factor model was built to test the main effect of the interaction effect of genotype and diagnosis, with mean framewise displacement (FD)^48^, gender, age, cohort used as covariates. Post hoc t tests were performed to further investigate the effect of genotype in different diagnostic groups (ADHD or control). The cluster-level analysis threshold was set to *P* < 0.01 determined by Monte Carlo simulation correction which was also utilized for multiple comparison correction, and recalculated the kernel of smoothness.

#### Expression quantitative trait loci (eQTL)

To explore the affected gene expression, we got the SNPs in LD with the significant SNP (*r*^*2*^ > = 0.75) using the 1000 Genomes Project ASN population data. The regulatory features related with these SNPs were searched in rVarBase^49^, HaploReg^50^ and RoadMap WashU EpiGenome Browser^51^. The eQTL data were searched in GTEx Portal^52^, SCAN^53^, seeQTL^54^, SMRI human prefrontal cortex eQTL data^21^, and BRAINEAC (http://caprica.genetics.kcl.ac.uk/BRAINEAC/).

### Data availability

The datasets generated during the current study are available from the corresponding author on reasonable request.

### Ethic approval

The study was approved by the Institutional Review Board of Peking University Sixth Hospital and carried out in accordance with relevant guidelines and regulations. Written informed consent was obtained from parents of the ADHD probands and controls.

## Electronic supplementary material

Supplementary Material
