# Supplementary material for: Genetic variant for behavioral regulation factor of executive function and its possible brain mechanism in attention deficit hyperactivity disorder
Source: Sci Rep. 2018 May 16;8:7620. doi: 10.1038/s41598-018-26042-y (PMC5956073; doi:10.1038/s41598-018-26042-y)
Supplement: Supplementary file 1 — Supplementary Material [file 41598_2018_26042_MOESM1_ESM.doc]

**Genetic variant for behavioral regulation factor of executive function and its possible brain mechanism in attention deficit hyperactivity disorder**

Xiao Sun^1^, Zhaomin Wu^4^, Qingjiu Cao^1^, Ying Qian^1^, Yong Liu^2,3^, Binrang Yang^4^, Suhua Chang^2,5^*, Li Yang^1^*, Yufeng Wang^1^

**Supplementary Material**

**Supplementary Methods**

**fMRI data acquisition.** MRI scanning were performed on Siemens Trio 3-Tesla scanner in the State Key Laboratory of Cognitive Neuroscience and Learning, Beijing Normal University. With head snugly fixed with foam pads and a belt, subjects in a supine position were instructed to close their eyes while keep awake and avoid thinking about particular issues during the scanning. For rest state MRI scanning at the first stage recruitment, parameters including an echo-planar imaging (EPI) sequence with 33 axial slices, repetition time (TR) =2000 ms, echo time (TE) =30 ms, flip angle=90 degree, slice thickness/skip=3.0/0.6 mm, in-plane resolution=64x64, 240 volumes, were used. For the second stage recruitment rest state scanning, parameters including an echo-planar imaging with 33 axial slices, repetition time (TR) =2000 ms, echo time (TE) =30 ms, flip angle=90 degree, slice thickness/skip=3.5/0.7 mm, in-plane resolution=64x64, 240 volumes, were applied. Following this 8-minute rest condition scanning sequence, high-resolution T1-weighted structural images covering the whole brain were acquired, which lasted 8 minutes. In the first cohort, 176 (sagittal) slices were obtained (TE=3.45ms, TR=2530ms, Flip angle=7 degree, Slice thickness=1mm, Slice Spacing=1mm, Matrix =256×208, FOV Size =256×208mm). At the second cohort, 128 (sagittal) structural images were acquired (TE=3.39ms, TR=2530ms, Flip angle=7 degree, Slice thickness=1.33mm, Slice Spacing=1.33mm, Matrix=256×192, in-plane resolution=1×1mm, FOV Size =256×256mm).

**fMRI data preprocessing and quality control.** Free software Data Processing Assistant for Resting-State fMRI (DPARSF[^1^](#_ENREF_1), http://rfmri.org/DPARSF), which is integrated by DPABI (<http://rfmri.org/DPABI>), was performed to progress image the preprocessing, including slice timing, realignment, reorientation, within-subject registration of rest fMRI and T1 image, nuisance regression, normalization, band-pass filtering (0.01-0.08Hz) and smoothness. DPARSF is developed based on Statistical Parametric Mapping (SPM12, http://www.fil.ion.ucl.ac.uk/spm/software/spm12/) and Resting-State fMRI Data Analysis Toolkit (REST[^2^](#_ENREF_2), http://www.restfmri.net). Quality control was applied with DPABI Quality Control toolbox. The quality of T1 and rest-state MRI data was checked before pre-processing. Any individual images failed to cover 95% the average individual whole-brain mask was excluded. Individual mean framewise displacement (FD) was used to calculate head motion derived with Jenkinson's relative root mean square (RMS) algorithm[^3^](#_ENREF_3). The threshold motion was set to 0.2. Any individuals failed the threshold and/or head motion over 3.0 mm and/or 3 degrees were excluded.

For the rest condition images, first ten time points were discarded for individuals to adapt the circumstance and scanner calibration. Slice timing was then used to correct the differences in signal acquisition time between slices. The acquisition of the slice at the mid-point of TR was set as the reference slice for shifting the signal measured in each slice. The time series of scans obtained from each individual were realigned with a 6 parameter (rigid body) liner transformation. All the scans are registered to the first image and then the mean of the images at the second pass procedure to improve the results. For better co-registration, individual structural images were skull-stripped and then co-registered to the mean functional image after realignment with a rigid-body transformation. An extended segmentation algorithm was used to segment the transformed structural images into gray matter (GM), white matter (WM) and cerebrospinal fluid (CSF). An algorithm for diffeomorphic image registration (DARTEL) tool was processed to evaluate transformations from individual native space to MNI space. Friston 24-parameter model was utilized to regress out head motion effects. Respiratory, cardiac effects and low-frequency drifts linear trends were reduced by regressing out WM and CSF signals and linear trend, respectively. The standardization of global signal regression (GSR) was also applied. Temporal filtering (0.01–0.08 Hz) was performed on the time series. After nuisance covariates regression, the functional volumes were spatially normalized to the MNI space in the use of the normalization parameters evaluated in DARTEL. Individual ReHo map was generated by calculating the Kendall coefficient of concordance (KCC) of the time series of a given voxel with those of its neighbors (26 voxels) in a voxel-wise way. Image volumes were smoothed with a Gaussian kernel of 4*4*4 mm full-width at half maximum (FWHM) after generating ReHo map. Average individual whole-brain mask was adopted to for further analysis and any voxels out of brain of participants was set to zero.

**Figure S1** The component plot in the rotated space and rotated component matrix for the normalized scores of eight components of BRIEF. The principle component analysis (PCA) was conducted using SPSS. The phenotype values of the eight phenotype values were normalized before the PCA.

**
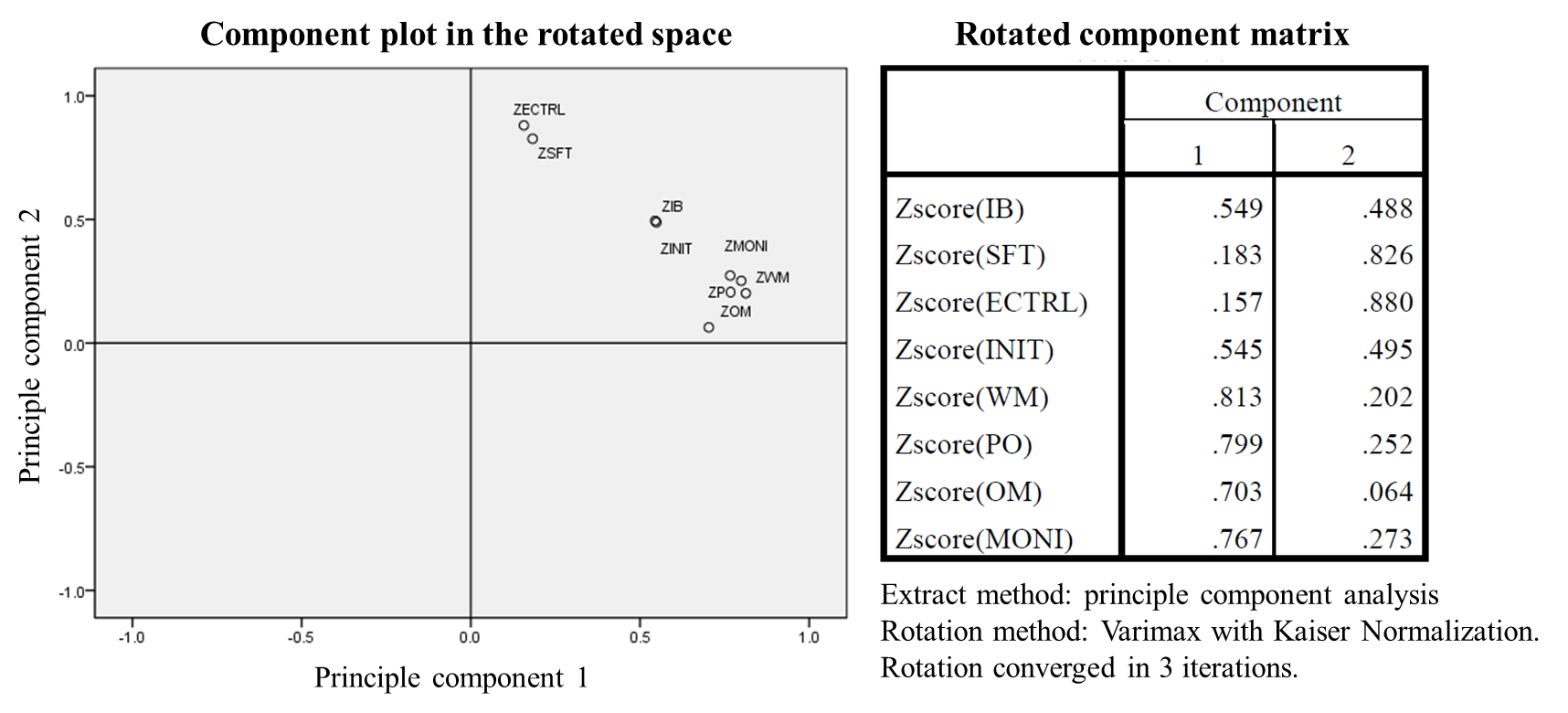
**

**Figure S2** Candidate 2-factor models for the confirmatory factor analysis and fit parameters for each model was shown below the model.

**
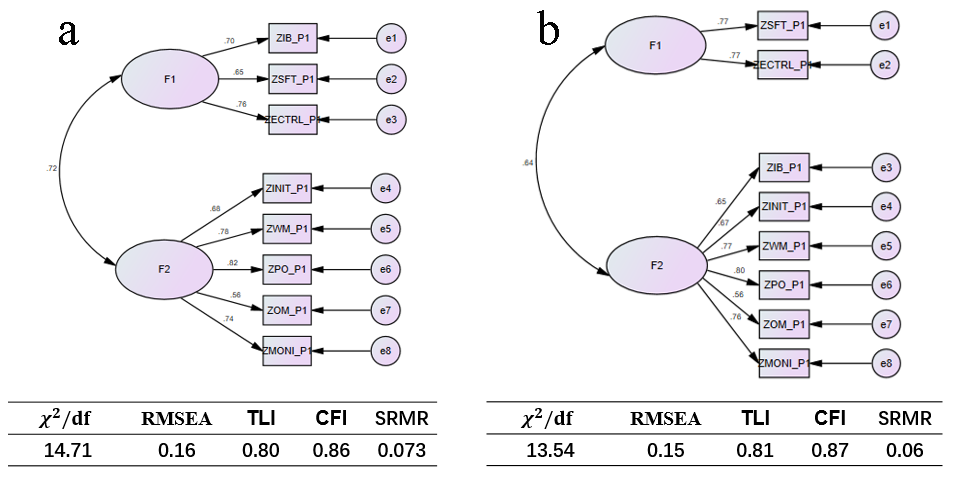
**

**Figure S3** Chromatin state near the significant locus rs852004.


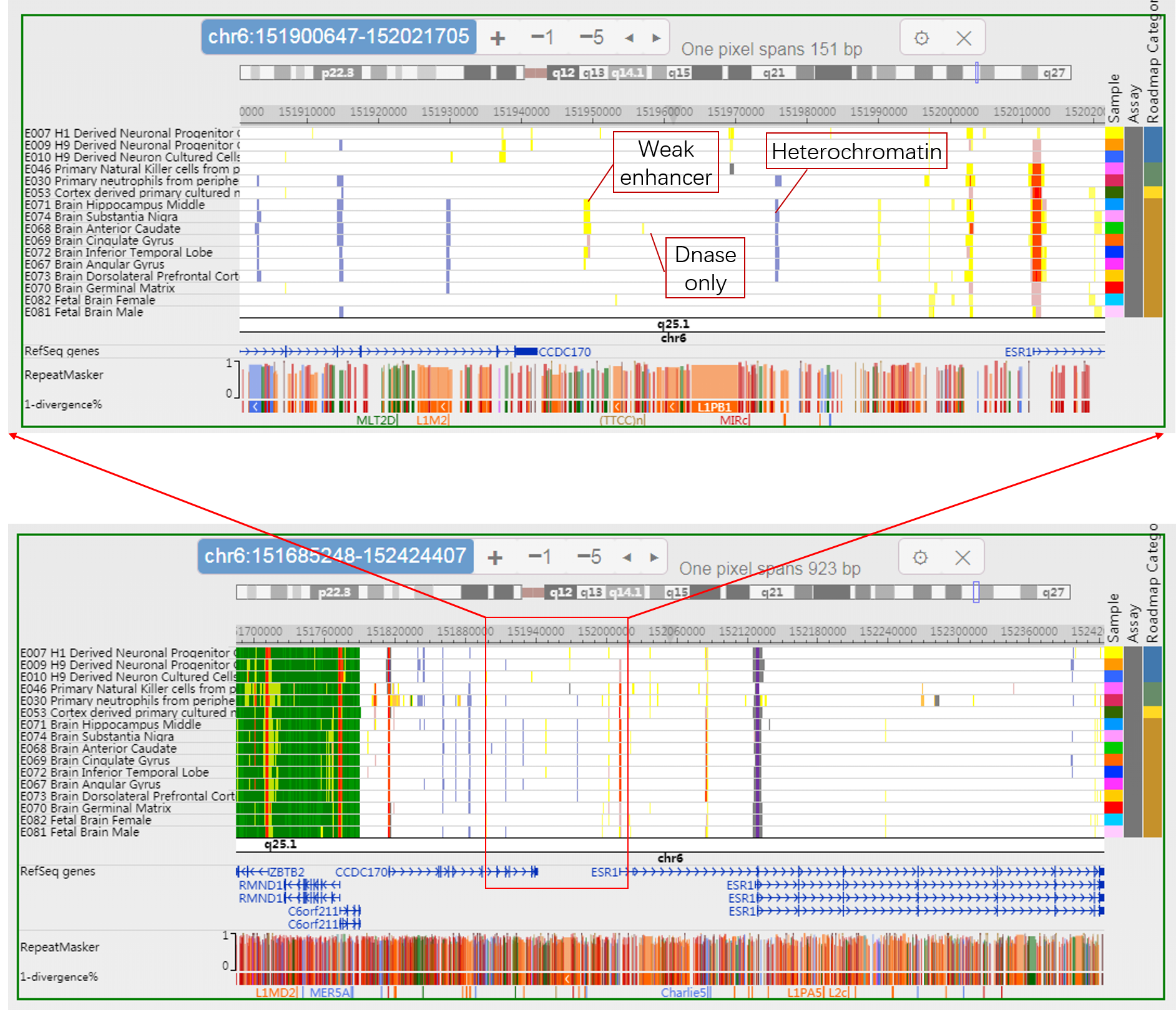


**Table S1** Indirect effect of rs852004 on ADHD symptom total score through each BRIEF scale as mediator.

|  | **Effect** | **Boot SE** | **Boot LLCI** | **Boot ULCI** |
| --- | --- | --- | --- | --- |
| **IB** | **0.9716** | 0.1654 | 0.6618 | 1.3019 |
| **SFT** | 0.1132 | 0.0578 | 0.0252 | 0.2563 |
| **ECTRL** | 0.2978 | 0.0901 | 0.1469 | 0.5077 |
| **INIT** | 0.1994 | 0.0756 | 0.0789 | 0.3865 |
| **WM** | **0.3795** | 0.1120 | 0.1893 | 0.6259 |
| **PO** | **0.3318** | 0.0947 | 0.1758 | 0.5528 |
| **OM** | 0.2418 | 0.0879 | 0.0769 | 0.4255 |
| **MONI** | **0.5910** | 0.1374 | 0.3392 | 0.8971 |

SE: standard error, LLCI: lower limit confidence interval, ULCI: upper limit confidence interval.

**References**:

1. Chao-Gan, Y. & Yu-Feng, Z. DPARSF: A MATLAB Toolbox for "Pipeline" Data Analysis of Resting-State fMRI. *Frontiers in systems neuroscience* **4**, 13 (2010).

2. Song, X.W.*, et al.* REST: A Toolkit for Resting-State Functional Magnetic Resonance Imaging Data Processing. *Plos One* **6**(2011).

3. Jenkinson, M., Bannister, P., Brady, M. & Smith, S. Improved optimization for the robust and accurate linear registration and motion correction of brain images. *Neuroimage* **17**, 825-841 (2002).
